# Addressing Barriers Newcomer Families Face When Obtaining Routine Childhood Vaccines in Alberta, Canada

**DOI:** 10.3390/vaccines12121380

**Published:** 2024-12-07

**Authors:** Siobhan M. Wong King Yuen, Emily J. Doucette, Caitlin Ford, Madison M. Fullerton, Ginamaria Vetro, Amanda Koyama, Jia Hu, Cora Constantinescu

**Affiliations:** 1 Department of Pediatrics, Cumming School of Medicine, University of Calgary, Calgary, AB T2N 1N4, Canada; 2 19 to Zero Inc., Rocky Mountain House, AB T4T 1B1, Canadagina.vetro@19tozero.ca (G.V.);; 3 Cumming School of Medicine, University of Calgary, Calgary, AB T2N 1N4, Canada; 4 Calgary Catholic Immigration Society, Calgary, AB T2R 0G5, Canada; 5 Department of Community Health Sciences, Cumming School of Medicine, University of Calgary, Calgary, AB T2N 1N4, Canada; 6 Pediatric Infectious Diseases, Department of Pediatrics, Cumming School of Medicine, University of Calgary, Calgary, AB T2N 1N4, Canada

**Keywords:** routine childhood vaccines, newcomer, immunizations, access, equity, qualitative methods

## Abstract

**Background/Objectives**: As the newcomer population in Canada continues to grow, we aimed to collaborate with newcomer families arriving in an urban center in Alberta, Canada to identify strategies to overcome identified barriers newcomers face in obtaining routine childhood vaccines (RCVs). **Methods**: We recruited newcomers living in Calgary, Alberta to participate in a workshop utilizing the Nominal Group Technique (NGT) to develop solutions addressing barriers to obtaining RCVs. Ranking exercises helped identify the top-proposed interventions based on perceived impact and feasibility for implementation. Based on the identified need for translated vaccine resources, infographics on school-based vaccines were developed. The infographics were pilot-tested in a first-language focus group before the final product was translated into 10 different languages. **Results**: Consensus from 15 NGT workshop participants identified five key solutions to facilitate obtaining routine childhood immunizations: (1) Increasing access to reliable vaccine information; (2) Ensuring vaccine information and healthcare services are available in different languages; (3) Increasing vaccine appointment availability and optimizing the booking system for ease of navigation; (4) Increasing the role of family doctors in vaccine counseling and administration; (5) Streamlining vaccine record tracking. We developed infographics on the vaccines children in Alberta can receive through school-based vaccine programs and these were pilot-tested with 16 participants in a first-language (Arabic) focus group. **Conclusions**: The collaborative and iterative process of solution development with newcomers provided a platform for knowledge translation through the development of educational resources on school-based vaccines, addressing the information barrier that newcomers identified when accessing RCVs.

## 1. Introduction

The benefits of childhood vaccines have been well established. Immunizations have significantly reduced morbidity, disability, and mortality against severe infectious diseases at both individual and population levels [1,2,3]. Despite these benefits, childhood immunization rates in Canada remain suboptimal [2] and recent polling has demonstrated that parental opposition to vaccinating their children is on the rise, particularly in Alberta, Canada [4].

Newcomers, including immigrants, refugees, and temporary foreign workers, face unique challenges in obtaining routine childhood vaccines, which contributes to lower vaccination rates amongst newcomer families [5,6]. Charania et al. completed a scoping review of published literature assessing immunization coverage among migrants worldwide [7]. Their review suggests that newcomer populations, including both immigrant and refugee groups, generally experience a higher burden of vaccine-preventable disease and lower immunizations rates post-migration compared to the general population of their host country [5]. Language barriers, cultural competency, and difficulties accessing and utilizing healthcare were a few of the complex factors which decreased vaccination rates and increased the burden of vaccine-preventable diseases within newcomer populations [5]. In Canada, newcomers represent approximately a quarter (23%) of the population [8] with the projected plan from Immigration, Refugees and Citizenship Canada (IRCC) to welcome between ~365,000 and 390,000 new immigrants per year between 2024–2026 [9]. While newcomers to Canada have initially reported to be in better health compared to local-born counterparts, this “healthy immigrant effect” does not apply to selected health conditions such as infectious diseases [10]. Considering the vulnerability of this group, the decreasing rate of immunizations in Canada, and access issues faced by newcomers, it is imperative that we understand barriers and implement solutions for newcomer families to access routine childhood vaccines as part of their transition to life in Canada.

In a previous study conducted in 2022, our research team held several focus groups with 47 newcomer parents in one rural and two urban settings within Alberta, Canada [11]. Although the majority of individuals acknowledged the benefit of routine childhood vaccines, the participants identified five key barriers that newcomer families face in getting their children vaccinated, including: (1) a lack of reputable resources for routine childhood vaccines, (2) language barriers when looking for information and asking questions about routine childhood vaccines, (3) difficulty accessing a family physician, (4) lack of affordable and convenient transportation options to attend vaccine appointments, and (5) lack of available vaccine appointments [11]. It should be noted that within Alberta—unlike other Canadian provinces—family physicians do not administer routine childhood vaccines; instead, they are administered by separate appointments at public health clinics, through school-based vaccine programs, and some vaccines are available via pharmacies.

The purpose of this study was to collaborate with newcomer families arriving in an urban center in Alberta (Calgary) to identify strategies to overcome the identified barriers newcomer families face in obtaining routine childhood vaccines. Based on the 2021 census, Calgary had the third highest proportion of immigrants among Canada’s 41 largest urban centers with foreign-born individuals making up 31.5% of Calgary’s population and 1.9% being non-permanent residents [12] and in 2023, Calgary welcomed the most migrants per capita than any other Canadian city [13]. We used the Nominal Group Technique (NGT) as a structured method of idea generation, providing a platform for discussion and debate, and gauging participant consensus to generate ideas. An advantage of the NGT is that this methodology equally considers the views of all participants, avoiding one individual dominating the group, and has demonstrated validity [14,15]. An added benefit of the NGT method is its time efficiency, cost-effectiveness, and adaptability. Using the information gathered from the NGT, we then sought to provide knowledge translation through the development of educational resources on school-based vaccines. Ultimately, this project lays the foundation of a larger project goal, which is to increase newcomer acceptance, knowledge, and confidence in Canada’s routine vaccine program.

## 2. Materials and Methods

**Figure 1:**
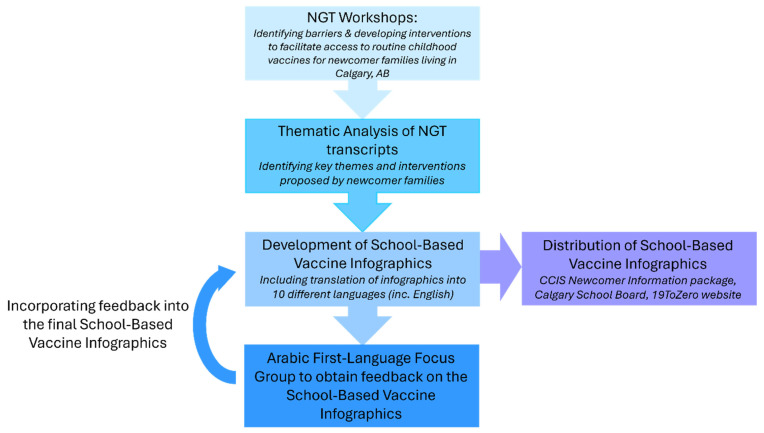
Overview of study methods.

Our study involved several different steps, including Nominal Group Technique (NGT) workshops for newcomer families, qualitative analysis, iterative development of vaccine infographics, and distribution of the resources to various community partners. Each step is described below and summarized in Figure 1.

### 2.1. Nominal Group Technique (NGT)

**Figure 2:**
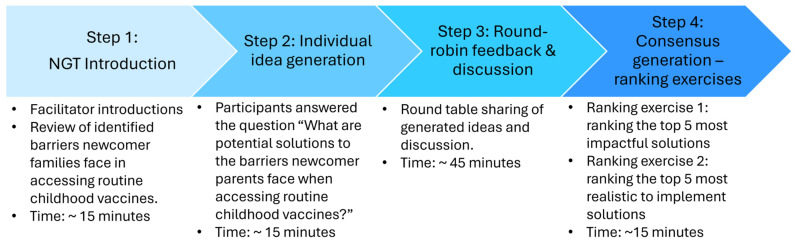
NGT workshop outline.

Our NGT sessions were adapted from the original NGT methodology [16] which consists of four steps: (1) Silent generations of ideas in writing, (2) Round-robin feedback from group members to record each idea, (3) Discussion of each recorded idea for clarification and evaluation, and (4) Individual voting on priority ideas (Figure 2). At the start of our workshop, facilitators gave a brief presentation to review the previously identified barriers that newcomer parents face in obtaining routine childhood vaccines as well as our project objectives. Next, participants were given 15 min to individually write down as many ideas to answer the following question: “What are potential solutions to the barriers newcomer parents face when accessing routine childhood vaccines?”. Following individual generation of ideas, 45 min were dedicated to Round-robin feedback and discussion of each idea. The last part of our workshop consisted of participants ranking the solutions into the five most impactful and the five most realistic to implement solutions. The workshop time frame was two separate 1.5-h sessions (3 h total), completed over 2 days. Following the NGT workshops, the facilitators debriefed and shared field notes.

### 2.2. Nominal Group Theory Participant Recruitment

Two workshops utilizing NGT methodology were held on October 2nd and 4th, 2023 with a total of 15 newcomer individuals in Calgary, Alberta, Canada. Participants were recruited through the Calgary Catholic Immigration Society (CCIS) [17], a non-profit organization providing both settlement and integration services to newcomers in Southern Alberta. Participants were asked to attend an in-person workshop to develop solutions to ease the identified barriers accessing routine childhood vaccines in Calgary. To be eligible to participate, participants needed to be newcomers to Canada (including refugees, immigrants, and migrant workers) who migrated to Canada within the last five years, had at least one child 18 years old or younger, and needed to be able to communicate in English. Participants received a $50 honorarium each for their participation.

### 2.3. Qualitative Analysis

The NGT workshops were audio recorded and recordings were transcribed verbatim to support thematic analysis. Two qualitative researchers conducted in-depth iterative thematic analysis following Braun and Clark’s 6-step framework [18]. NVivo Qualitative Data Analysis Software (QSR International, Version 14), a qualitative data analysis software, was used to organize data and facilitate analysis. Coding and thematic analysis were also supported by reviewing field notes recorded during each workshop. Regular communication between the data analysts ensured that final themes were agreed upon. In the discussion of themes, quotations from participants were provided along with their participant number, gender, and country of origin (Supplemental Data, Tables S1 and S2).

### 2.4. Ranking Exercises

Following the NGT discussion, participants were asked to rank the proposed interventions into the top five interventions that would provide the greatest impact in facilitating access to routine childhood vaccines for newcomer families and the top five interventions that were the most feasible to implement. Thirteen out of fifteen participant responses could be analyzed for the top five most impactful interventions while ten out of fifteen participant responses could be included in the analysis for the top five most feasible interventions. Excluded responses were those who were unable to complete the exercises correctly (e.g., no response to this exercise).

### 2.5. Development of School-Based Vaccines Infographics

Our group of researchers (including a pediatric resident at the Alberta Children’s Hospital, researchers at 19 to Zero [19], and members of the CCIS team developed infographics on the vaccines children in Alberta can receive through school-based vaccine programs. In Grade 6, children receive the hepatitis B and human papillomavirus (HPV) vaccines and in Grade 9, diphtheria, tetanus, acellular pertussis (dTap) and meningococcal (Groups A, C, Y, W-135) conjugate vaccines. Vaccine information was obtained from resources available from Alberta Health Services [20] and our research team used an iterative process to distill information to the key elements for our infographics. The infographics were then translated into nine different languages (Arabic, French, Mandarin, Punjabi, Spanish, Tagalog, Tigrinya, Ukrainian, and Vietnamese), based on the demographic information of the most populous minority groups served at CCIS. Additionally, stakeholders with the Calgary Board of Education provided logistical advice for how to distribute the infographics to newcomer families within the Calgary school system.

### 2.6. School-Based Vaccine Infographic Focus Group

Following the development of the infographics, a one-hour first-language (Arabic) focus group was chosen as a case study with 16 participants recruited from CCIS to pilot-test the school-based infographics and gain feedback. Arabic is the fifth most spoken non-official language in Alberta [21] and given the large proportion of Arabic-speaking clientele served by CCIS, conducting a focus group in Arabic was the most feasible to complete in the time constraints of this study. The focus group started with a brief overview of the school-based vaccine program in Alberta. Most of the focus group was spent obtaining feedback on the clarity, design, relevance, and persuasion of the developed infographics, which was then used to improve the design and content of the infographics. CCIS interpreters were present during the focus group and were instrumental in helping our research team analyze feedback from the focus group. A final iteration of adjustments to the infographics was made based on feedback received during the newcomer first-language focus group.

## 3. Results

Two 1.5-h NGT workshops were held with 15 participants, representing 10 countries of origin (countries that participants had migrated to Canada from) (Table 1). Overall, the participants included ten females and five males, who had been in Canada between 6 months to 5 years and had between 1–3 children aged from >1 year to <18 years old per family. The age of participants was between 28–45 years.

### 3.1. Identified Barriers Newcomer Families Experience in Obtaining Their Children’s Routine Childhood Vaccines in Calgary, Alberta, Canada

During our workshops, five key barriers were identified by newcomer families when accessing routine childhood vaccines: (1) Difficulty navigating the appointment book system and lack of flexibility in appointment availability; (2) Lack of reliable and easy-to-access transport to attend vaccine appointments; (3) Language barriers during vaccine appointments and a lack of vaccine information in home language; (4) Rise in anti-vaccine rhetoric and vaccine conspiracy theories, including a lack of trustable vaccine information to help parents make vaccine decisions; (5) Difficulty accessing a family doctor for vaccine information and obtaining vaccines (Table S1).

### 3.2. Key Solutions Newcomer Families Identified to Facilitate Obtaining Their Children’s Routine Childhood Vaccines

Parents identified five key areas, that if addressed, would facilitate routine vaccinations for their children: (1) Increasing access to reliable vaccine information; (2) Ensuring that vaccine information and healthcare service are available in different languages; (3) Increasing vaccine appointment availability, including utilization of community outreach programs, and optimizing the booking system for ease of navigation; (4) Championing family doctors to play a pivotal role in vaccine counseling and administration; (5) Streamlining vaccine record tracking.

### 3.3. Interventions

#### 3.3.1. Interventions 1 & 2: Increasing Access to Reliable Vaccine Information and Ensuring Resources Are Available in Different Languages

Parents expressed difficulties finding information about vaccines as a barrier to obtaining their children’s vaccines. Pertinent information that parents requested about vaccines included vaccine names, what disease(s) the vaccine protects against, ingredients contained within the vaccine, possible vaccine side effects, the vaccine schedule followed within Alberta, and the process for scheduling vaccines. Given that the Government of Alberta has the MyHealth Alberta website with the vaccine schedule and information [20], the focus of this intervention should be to educate newcomer families about this resource. Ensuring that families know how to access this website can be facilitated through their first medical appointment in Canada and public health can also direct families to this online resource.

Parents identified the need for healthcare services to be accessible in different languages. Parents discussed the difficulty and hesitancy they had around booking immunizations via phone with public health clinics given that translation services were not readily available. Thus, ensuring that interpreters are available during the vaccine booking process and during vaccine appointments will give newcomer families the confidence to participate in their children’s immunizations. Additionally, ensuring that vaccine-related resources are available in different languages was also identified as a key factor in ensuring parents are well-informed about their children’s vaccines.

Families thought distributing vaccine information at daycares and schools, at family doctors’ offices and hospitals, at community and religious centers, and as part of newcomer orientation packages would be a helpful start to easing this barrier and empowering families with vaccine knowledge. Newcomer parents who had children born in Canada also identified that distributing vaccine information as part of the newborn information package would be beneficial as well, especially since most routine childhood vaccines are scheduled for infants and toddlers.

Vaccine information sessions were seen as a further step to help bridge the information gap about routine childhood vaccines and facilitate the process of obtaining vaccines. Parents were open to information sessions held at schools/daycares and community organizations, and even coordinated with IRCC pre-immigration workshops as helpful tools to facilitate immunizations once families have arrived in Canada. These information sessions were seen as an important link that families could establish with the Canadian healthcare system.

Families identified that the responsibility for knowing when their child was due for immunization was largely on them. Thus, developing an automated vaccine reminder system (via email/online) would help reduce the onus on families and prevent delays in their children receiving their scheduled vaccines. Following vaccine appointments, parents also would like a physical document to remind them when their child’s next vaccine is due.

Additionally, families expressed hesitation when making decisions about seasonal vaccines (e.g., the annual influenza vaccine and COVID-19 vaccine) and thought having more information would guide their decision-making process.

#### 3.3.2. Intervention 3: Improving Availability and Access to Vaccine Appointments

Parents voiced challenges attending vaccine appointments during working hours as it often meant that they needed to take time off work to attend these appointments. Although several public health clinics around the city of Calgary offer 1–2 evenings per week with extended clinic hours, parents strongly advocated for more extended evening clinic hours as well as weekend clinic appointments to reduce the burden of having to take time off regular commitments to attend their children’s immunization appointments.

Families who do not have easy access to a personal vehicle noted transportation difficulties in attending the public health clinics. Transportation challenges were amplified when traveling with young children, in a new environment, and during winter conditions. In response to this need, many parents also identified that increasing the number of public health clinics around the city would facilitate access to immunizations.

Families were in favor of utilizing daycares, schools, local places of worship, and community organizations (such as cultural centers or newcomer centers) as places to obtain vaccine information as well as administer vaccines. Newcomer families described that their local community centers or religious centers were one of the first organizations that they affiliated with upon immigrating to Canada. Thus, hosting vaccine drives at these local organizations was seen as a means to facilitate vaccine access to newcomer families. Given that children attend daycare and school daily, parents also thought these settings would be ideal for vaccine drives for their children’s routine immunizations.

Families identified challenges in booking their children for vaccines, thus developing a strategy to facilitate the vaccine booking process was a key intervention identified. Parents thought that a web-based booking system or a phone app would be helpful and the most accessible. Ensuring that these resources can be accessed in different languages would be key as well. If the booking system could be combined with vaccine information that would be the most ideal.

A key challenge that parents identified was needing to remember when their children were next due for immunization. A solution that was proposed by workshop participants was having “fixed open dates” for immunizations, set days where families can get their immunizations either at public health clinics without an appointment or vaccine drives at community organizations.

Families acknowledged that there is a general shortage of healthcare providers which places a strain on the healthcare sector and thus may limit extending public health appointments into the evening and over weekends. A solution that was proposed was utilizing foreign-trained healthcare workers to help staff immunization clinics. Although participants were cognizant of regulations healthcare professionals need to undergo to be able to practice within Canada, they thought immunizations were routine procedures whose practice variability from country to country was minimal and thus foreign-trained practitioners would be able to administer vaccines.

#### 3.3.3. Intervention 4: Championing Family Doctors to Play a Pivotal Role in Vaccine Counseling and Administration

Parents compared the role that family doctors play in managing children’s routine immunizations from their home country to the current practice that they have witnessed since they immigrated to Canada. Several parents recounted that family doctors played a much larger role in vaccine counseling and administering vaccines in their home country and reflected that they would like to see a similar role of family doctors in the immunization process in Calgary. Families reflected that family doctors were well positioned to provide counseling on their children’s vaccine schedule, provide reminders for when vaccines were next due, and provide counseling on which vaccines are considered seasonal.

Families also voiced that family doctor clinics and walk-in clinics were more numerous throughout the city and thus easier to access compared to the sparser public health clinics. Thus, if family doctors could administer vaccines, parents thought this would ease transportation challenges and would increase flexibility to be able to obtain their children’s routine immunizations.

#### 3.3.4. Intervention 5: Streamlining Vaccine Record Tracking

Several families shared their impression that their children had received multiple duplicate vaccines upon immigration to Canada given that there was a lack of vaccine record sharing between different countries/health agencies. Parents expressed frustration that vaccine names differed between countries and felt strongly about the development of a vaccine equivalency table to facilitate record keeping and ensuring that their children get the right dosing of vaccines needed for immunization, regardless of the country in which they had received their vaccines. Families thought that a vaccine equivalency table would also be beneficial to healthcare agencies to determine which vaccines individuals had received despite different vaccine brand names.

Parents also advocated for access to an electronic vaccine passport or electronic certificates to facilitate vaccine record keeping, especially given that parents felt the burden of remembering when their children were due for vaccines was largely their responsibility. Although the infrastructure is in place for electronic vaccine passports in Alberta, more resources should be invested in helping families navigate these vaccine passports.

### 3.4. Ranking Exercise

To conclude our focus groups, participants were asked to rank the top five solutions proposed based on how impactful the intervention would be in facilitating newcomer family access to routine childhood immunizations (Table 2). Increasing access to vaccine resources, including increasing access to vaccine information via physical paper information or web-based platforms, and having vaccine information available in multiple resources was seen as the top solution to facilitate vaccination among newcomer families. The next most impactful strategy proposed was the development of global vaccine equivalency tables which would facilitate vaccine record keeping between different countries. Increasing immunization appointment availability was the third most impactful solution ranked by participants. Participants wished to see extended hours of vaccine appointments (late evening and weekend appointments), as well as increased public health clinics around the city of Calgary. Increasing the role of family doctors in administering vaccines and providing vaccine counseling was ranked as the fourth most prioritized solution for participants, including utilizing walk-in clinics for vaccine administration. Increasing parental access to vaccine records (via physical paper copies of immunizations and via electronic records) was the fifth most highly ranked intervention proposed to facilitate vaccine access to newcomer families.

In terms of the most feasible to implement interventions, most participants thought that increasing access to vaccine resources was the most realistic method of increasing access to vaccines for newcomer families. Creating a global vaccine equivalency table was ranked as the second most feasible intervention, followed by increasing public health clinic availability as the third most feasible intervention. Facilitating vaccine record-keeping for parents and increasing the role of family doctors were rated as the fourth and fifth most realistic interventions to increase newcomers’ access to vaccines, respectively.

### 3.5. Developing School-Based Vaccine Infographics

**Figure 3:**
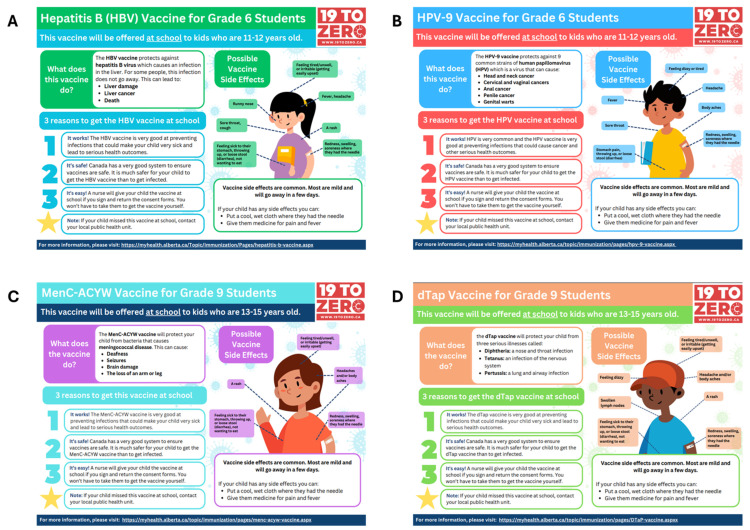
School-based vaccine infographics: (**A**) Hepatitis B (HBV) vaccine; (**B**) HPV-6 (human papillomavirus) (**C**) MenC-ACYW meningococcal; (**D**) dTAP (diphtheria, tetanus, pertussis) vaccine information sheets.

Given that families identified a lack of vaccine information as a barrier to getting their children immunized, this was a tangible starting point to facilitate vaccine decision-making for newcomer parents. Parents identified uncertainty with regard to the age when their children should be receiving their routine childhood vaccines, a lack of clarity regarding the diseases that the vaccines prevented and possible vaccine side effects. To fill this knowledge gap, our research group developed infographics targeting school-based vaccines (Figure 3). School-aged children receive the Hepatitis B (HBV) and HPV-9 (human papillomavirus) vaccines in Grade 6 and the MenC-ACYW (meningococcal) and the dTAP (diphtheria, tetanus, and pertussis) vaccines in Grade 9. The infographic sheets were translated into nine different languages, including Arabic, French, Mandarin, Punjabi, Spanish, Tagalog, Tigrinya, Ukrainian, and Vietnamese. Including English, these were the top 10 languages spoken by newcomer families within Calgary as identified by CCIS. Based on the Arabic first-language focus group feedback, participants conveyed that maximizing visual presentation with bullet-point information was the preferred manner of relaying information and this was highlighted in our final product.

## 4. Discussion

In this study, we used the NGT to conduct workshops with newcomer families to identify barriers they face when accessing routine childhood vaccines, generate potential solutions to the barriers, and use the findings to inform the development of school-based vaccine infographics alongside researchers, healthcare workers, and community partners. The vaccine infographics were then pilot-tested during a first-language focus group with a small group of newcomer parents who provided feedback that was incorporated into the final version of the resources.

From the NGT workshops, we identified five barriers that newcomer families face in regard to immunization services in Alberta, including difficulty navigating the appointment booking system, lack of reliable access to transport to attend appointments, language barriers during appointments and lack of vaccine information in the home language, a rise in anti-vaccine rhetoric and vaccine conspiracy theories, and difficulty accessing a family doctor for vaccine information and obtaining vaccines. These findings are similar to those previously identified through focus groups conducted with newcomer families in Alberta, Canada by our research team [11], and have been identified in other communities worldwide [5].

Similar to our findings, a systematic review found that most immunization barriers can be separated into access-related barriers and acceptance-related barriers (including knowledge and information barriers) [5]. As newcomers are more likely to immunize themselves and their children when vaccine access barriers are removed when compared with non-newcomer populations [22], awareness of these information barriers provides a key opportunity to improve vaccine uptake. Our research emphasizes the identified need for reliable vaccine information when families are deciding about immunizations for their children.

The goal of our study was to ensure our research outcomes would effectively address the barriers newcomer families face in obtaining routine childhood vaccines and it was imperative that newcomer families were involved in the process of generating potential solutions. The strength of this study includes the extensive engagement with the target population, the iterative process of solution-development with a strong pragmatic, and action-oriented component via the focus groups and ranking exercises. The solution with the greatest potential impact and the most feasible solution based on participants’ ranking was to increase access to reliable vaccine information. It has been shown that newcomers are more likely to trust health information received from healthcare providers and official sources such as community organizations and newcomer groups [23,24,25]. Given this information and the results of the focus groups and ranking exercise, we designed several one-page resources to provide key information on four routine childhood vaccines which children receive as part of the school-based immunization program within Alberta, and these resources were translated into nine different languages. The goal of creating these resources was to have them accessible online through government and health services websites, available in family physician offices, provided to newcomers in welcome packages from community groups, and distributed to parents via the Calgary school board.

The key strengths of this project include the co-development of vaccine resources based directly on the identified needs and solutions of newcomers who participated in the NGT workshops and collaboration with a multidisciplinary group of researchers, healthcare workers, and community partners. Further, the iterative process of resource development allowed for additional feedback from newcomers to be incorporated prior to dissemination. By conducting a first-language focus group, we have aimed to make our research more accessible and inclusive to members of society who may not traditionally participate in research activities due to language barriers. The infographics are also available in nine different languages, making them more likely to be used by newcomers than previous resources available in English only. This is also very timely work, as migration has steadily increased over the last few years worldwide and this helps in healthcare preparedness for host countries such as Canada.

The small number of NGT workshops conducted and recruitment from a single community organization may present a limitation to the overall generalizability of our findings. However, 2–3 workshops have been shown to successfully identify up to 80% of themes [26]. Given the narrow target population (newcomers to a Canadian urban center), the workshops were likely able to identify the most prominent themes as thematic saturation was met following analysis. Additionally, the NGT workshops were conducted in English only, which may have led to the exclusion of additional barriers faced by newcomers who do not speak English. The first-language focus group was conducted in Arabic with only one cultural group meaning they required translators and interpreters for analysis. This resulted in methodological challenges for the English-speaking research team, including the inability to use direct quotes. Given the time constraints of the study, only one first-language focus group was conducted to confirm the resources adequately addressed the concerns of the population prior to dissemination. Further research will be needed to confirm our findings and more thoroughly evaluate the effectiveness of the infographics in English and the nine additional languages the infographics were translated into.

## 5. Conclusions and Future Directions

To date, the infographics have been shared with several partners across Canada in healthcare, education, and community service sectors to support meaningful engagement with newcomers around immunizations. While it is too early to evaluate the reach and impact of the resources on newcomer immunization rates, it can be expected that the resources will contribute to newcomer families feeling more knowledgeable about school-based vaccines, and more confident in making informed decisions for their children.

By creating tailored educational resources that have been disseminated through pre-existing information pathways with our community partners, we have started to address one of the several barriers experienced by newcomers when accessing information about immunizations in Canada through the school-based immunization programs. It is important to note that other access-related barriers, including a lack of transportation to clinics and inaccessible vaccine booking systems, that were identified are not addressed in this study. Table 3 assesses each of the interventions proposed by newcomer families and provides suggestions for how these can be implemented into practice. Further health systems research should be conducted to address these additional barriers, which can be aided by involving newcomers and community partners in the development of future resources.

## Figures and Tables

**Table 1:** Participant demographics.

**Age, mean (SD), range in years**	34.6 (4.6), 28–45
**Gender: n (%)**	
Female	10 (67%)
Male	5 (33%)
**Average number of children**	1.6
**Average age of children (years)**	3
**Average number of years in Canada**	1.5
**Country of origin, n (%)**	
Algeria	1 (6.7%)
Brazil	1 (6.7%)
Eritrea	1 (6.7%)
Hungary	1 (6.7%)
India	6 (40%)
Lebanon	1 (6.7%)
Nigeria	1 (6.7%)
Pakistan	1 (6.7%)
Sudan	1 (6.7%)
Ukraine	1 (6.7%)

**Table 2:** Top five most impactful interventions to increase the accessibility of routine childhood vaccines to newcomer families in Calgary, AB.

Ranking	Proposed Intervention
1.	Increasing access to reliable vaccine information
2.	Development of vaccine equivalency tables to facilitate vaccine record-keeping between different countries
3.	Increasing vaccine appointment availability and facilitating easier booking, including utilization of community outreach programs
4.	Championing the role of family doctors in vaccine counseling and administration
5.	Streamlining vaccine record tracking

**Table 3:** Suggestions for implementing proposed interventions into practice.

Proposed Intervention	Suggestions for Implementation
Increasing access to reliable vaccine information	• Expansion of the vaccine infographics for all routine childhood vaccines, which can similarly be included in newcomer orientation manuals • Distribution of vaccine resources to the offices of family doctors • Directing families to online vaccine material via their community centers (places of worship, cultural centers, schools, and daycares).
Ensuring that vaccine information and healthcare services are available in different languages	• Translating vaccine information and school immunization consents to the top 10 spoken languages within Calgary • Ensuring access to translators during the vaccine phone booking process • Ensuring access to translators during vaccine appointments with public health
Increasing vaccine appointment availability, including utilization of community outreach programs	• Engaging with vaccination centers to extend public health services into the evening and weekends and offer drop-in appointments • Utilizing physician trainees to host vaccine drives at community organizations • Utilizing community pharmacists to administer routine childhood vaccines
Championing family doctors to play a pivotal role in vaccine counseling and administration	• Distributing vaccine resources in the offices of family physicians • Expanding the role of allied health professionals (e.g., nurses, nurse practitioners) within the family physician offices in providing vaccine counselling and administration
Streamlining vaccine record tracking	• Development of an electronic passport to help track a child’s immunization record

## Data Availability

The deidentified data presented in this study are available upon reasonable request from the corresponding author.

## Supplementary Materials

The following supporting information can be downloaded at: https://www.mdpi.com/article/10.3390/vaccines12121380/s1, Table S1: Barriers identified by Calgary newcomer families when accessing routine childhood vaccines; Table S2: Interventions identified by Calgary newcomer families to facilitate access to routine childhood vaccines.
