# Supplementary material for: Addressing Barriers Newcomer Families Face When Obtaining Routine Childhood Vaccines in Alberta, Canada
Source: Vaccines (Basel). 2024 Dec 7;12(12):1380. doi: 10.3390/vaccines12121380 (PMC11680383; doi:10.3390/vaccines12121380)
Supplement: Supplementary file 1 [file vaccines-12-01380-s001.zip › vaccines-3321736-supplementary.pdf]

Addressing Barriers Newcomer Families Face When Obtaining Routine Childhood Vaccines in Alberta, Canada

Supplementary Data

Table S1. Barriers identified by Calgary newcomer families when accessing routine childhood vaccines.

Identified Barrier	Subthemes	Key Quotes
1) Difficulty navigating the appointment booking system and lack of flexibility in appointment availability	Difficulty navigating the appointment booking system	• *"[It's] difficult to schedule an appointment, it was hard to have to call like so many times, and like whenever they had an appointment, I couldn't make it."* - participant 11, male, Brazil
	Limited appointment dates and times available	• *"But until this time, my son was, he, uh, he should take them at two years old, he was two years and four months until he took the vaccines because when I called them, I told them, "I need an appointment after my work," and they have only like two days they open 'til 8:00… So, having this only two days opening until 8:00, all the people have their appointments on these two days."* - participant 15, female, Lebanon
2) Lack of reliable and easy to access transport to attend vaccine appointments	For families who do not have access to a personal vehicle, relying on public transit to attend appointments is challenging	• *"Transportation, 'cause you arrive here by the winter, no car, having to … take a small kid around train and walk around with it, with her, was like tough"* - participant 11, male, Brazil
3) Language barriers during vaccine appointments and lack of vaccine information in home language	Families struggle with the vaccine appointments when they are unable to speak/understand English and don't have access to a translator during the vaccine appointments	• *"I worked together with the lady at the AHS the other day, and like okay, that's this, that's this. Okay, so we've got somewhere, but it wasn't easy for her, like she didn't speak Portuguese, my English when I got here was not that good, so that's a barrier for us that just arrived here."* - participant 11, male, Brazil
	Obtaining service in French, Canada's second official, is challenging within Alberta	• *"...For instance, Francophones are, uh, struggling ... Even if it's like second language or it's first language of Canada, but it's, uh, still difficult to find someone that speaks French that can understand you, so without speaking about other languages… If we don't find services in French (laughing), how can we expect other language?"* - participant 14, female, Algeria
4) Rise in anti-vaccine rhetoric and vaccine conspiracy theories	Since the COVID-19 pandemic, families are hesitant to vaccinate their children due to the rise in vaccine conspiracy theories	• *"'Cause I know when I came here it was like 2021, and was still facing COVID, and back home … like a lot of diseases that were already like gone, they're starting to come back because moms and dads, they don't wanna give vaccines anymore. Mainly because of COVID and like conspiracy theories or whatever. So, I guess, information takes a good part on that … Bringing people back, like, "Okay, this is safe and this is what it does, we've been doing this safe for 30 years, and this is what happens and your kid is disease free." And I guess this goes for everyone, and having information I guess is the key point"*. - participant 11, male, Brazil
	Lack of trustable vaccine information to help parents make vaccine decisions is a limitation to vaccinating their children	• *"The lack of information. And I've noticed that even at the family doctor, she didn't have anything to give me. She said, "Oh, yeah, you call this number, and this number will let you know what you need to do." So she didn't have that like, okay, she's three years old, though, she needs this, this, this, this, this … I guess having that information at least on a website would kind of provide us with some guidelines to where to start from" ."* - participant 11, male, Brazil

5) Difficulty accessing a family doctor	Family doctors were seen as playing a key role in providing parents with vaccine information and obtaining vaccines	• *"We came to know where to go and get the vaccines only after meeting a family doctor, so until then we didn't know where to go … but getting the family doctor … also is tough"* - participant 7, female, India<br>• *"But I also know that not everyone gets a family doctor when they arrive, so that's another issue. I faced that, too."* - participant 11, male, Brazil
	Families identified challenges in securing a family doctor and this impacted obtaining routine immunizations for their children	• *"Initially I just came one year back, so in six months I don't have the family doctor"* – participant 5, female, India<br>• *"But I also know that not everyone gets a family doctor when they arrive, so that's another issue. I faced that, too."* - participant 11, male, Brazil

Table S2. Interventions identified by Calgary newcomer families for to facilitate access to routine childhood vaccines.

Intervention	Subthemes	Key Quotes
1) Increasing access to reliable vaccine information	Families advocated for the distribution of vaccine information at various community organizations, including daycares, schools, community and religious centers, as well as healthcare facilities like family doctors' offices	*"It [vaccine information] should be part of like the newcomer's package in terms of the orientation they are given, so, 'cause I understand that, uh, almost every newcomer, one way or the other, looks for one of these newcomer centers to identify with … Orientation on the vaccines for their kids should also be part of the teachings you give them to know how they can easily continue from where they start from home."* - participant 13, male, Nigeria
	For children born in Canada, families thought that vaccine information should be provided as part of the newborn information package prior to being discharged from hospital	*"If a baby got birthed in Canada, because my baby got birthed in Canada in this year, so a hospital where the baby is getting birthed they are giving you thousands of papers… So you can add two papers more to give that- what is all this vaccine? But they will tell you two months four months six months vaccine, but no one is telling what vaccine, because as agreed, uh, different countries have different vaccines."* - participant 8, female, Hungary
	Vaccine information sessions held at community organizations and even IRCC pre-immigration workshops was seen as a way to bridge the information gap about RCVs	• *"By organizing more sessions, more information session, workshops … through daycares, communication with daycare and parents, at school, at community associations, and whatever. Uh, just to make communication between the healthcare services and the parents easier."* - participant 13, male, Nigeria<br>• *"So, I guess on the immigration process that we go through… We have a lot of information about jobs, we have information about, "Oh, this is what you need to do when you get there," like you said about the welcome packages, but before you're even coming here, if you're coming with a kid, you need to know this….Because what kind of vaccination she needs to take before coming? What will she need to take when she comes? And we don't know that information, this is not told to us."* - participant 11, male, Brazil
	Development of an automated vaccine reminder system (via email/online) would help reduce the onus on families and prevent delays in their children receiving their scheduled vaccines	*"If there is some kind of automatic system that will get reminders through email or something, I'll do a vaccine and get vaccines when due, that would be good"* - participant 9, female, India
	Families identified a need for information about seasonal vaccines	*"I think for non-mandatory [seasonal vaccines] you have to … present [the information] - what are the side effects? So if there are side effects and all that, so then the parent should decide and then they can go for non-mandatory [seasonal vaccines]. So that information is not available."* -participant 5, female, India
2) Ensuring that vaccine information and health care service is available in different languages	The need for translators during vaccine phone booking appointments and during healthcare appointments was identified as a major intervention required to facilitate vaccine access	

	Families identified the need for vaccine information to be available in different languages	*"[A] website with all, like at least major languages that we have here in Calgary now. Like, uh, we have a lot of countries, people from different countries living here, but like I guess everyone kinda speaks English being here, so having at least in English … like technical names for the vaccines that we could kinda look into. 'Cause I know, at least in my country, when I look at the vaccine, it has the name in Portuguese, but also the substance that's there, like, uh, whatever chemicals".* - participant 11, male, Brazil
3) Increasing vaccine appointment availability, including utilization of community outreach programs, and optimizing the booking system for ease of navigation	Families strongly advocated for more extended evening public health clinic hours as well as weekend clinic appointments	*"Around here, I think most of the clinics will close by five o'clock or so, but for us it is difficult, like once we are working, we have to take a day off for that. So, mostly in my country, you know, they used to work 'til nine o'clock, so after my work we would go and take the vaccines."* - participant 12, unknown, unknown
	Increasing the number of public health clinics around the city would ease transportation burdens for families	*"Having more places that offers the vaccines would be great, 'cause not everyone can drive when they get here, they can't afford a car. And [taking] small kids over the train, bus, to places far away, it is not easy. Like, 'cause we don't know to walk around the city"* - participant 11, male, Brazil
	Vaccine drives held at community or religious centers, schools, and daycares was seen as an ideal solution to make vaccines more accessible to newcomer families	• *"Creating more centers for these vaccines, and to create these centers, we should make it more regional to the people. I can say categorically that the majority of the new immigrants are religious based, all right? All right, so they have one religious background or the other, and for the most part, before they even start working, they have identified with their religious bodies … I would advocate that vaccines should be brought to their religious places of worship. For example, in my country they even bring vaccines to your place of worship on Sundays … After the service, the father will announce it in the church, "children between the ages of this and this, you'll be taking the vaccine next week or next month, the people from the local government will be coming for that". And it makes it kind of easy, it eliminates that stress of booking appointments, that stress of looking for availability of the parents to come and everything. If you wanna make these vaccines available for them, we should be able to take it to them. For the most part, a lot of them do not begin to drive early enough. It's a challenge. A lot of them don't have jobs, and if only they have jobs, they've still got having to do multiple jobs, you know? So, it doesn't make it easy for them to make an appointment. So, when you take it to where they can easily meet up with, uh, their cultural groups, where they get emotional support, you know, comfort and everything, they will be able to access this easily."* - participant 13, male, Nigeria<br>• *"They [parents] would always come and pick the kids after work or drop them off, you know? It's also an easy access for them, so they may just use that time there to drop off the child, get the necessary information, fill the necessary documents, have the child vaccinated, and they come back to get the child. The same place of their daycare is the same place they are getting the vaccines, too. It's makes life a lot easier for them. So, I think that's a way the- the level vaccination increases."* - participant 13, male, Nigeria
	Developing a web-based or phone application-based booking system was identified as a key intervention to reduce challenges with booking vaccine appointments.	*"[A] website would be something easier, maybe even on the website having more tools there that you can schedule appointments. Phone apps, they're not that hard to do nowadays … like [the] user experience on that would be nice, that you can have like different languages, I don't know. Uh, like Arabic, or you can have English, you can have Spanish, like the main languages that we have here now, like most of the peoples, I think that would be one good idea to do."*- participant 11, male, Brazil

	Utilizing foreign trained healthcare providers to staff immunization clinics was a solution proposed	• *"I understand there's a regulation, they need to go over that, that's fine, I agree with that, but like for some procedures, which is actually giving a shot, is not... If they have the proper training back home…"* - participant 11, male, Brazil<br>• *"Cause I know I've went to- to, like the- the immunization center, they don't have a lot of people working there, I- I knew I had to wait like two hours or whatever because there was only one person doing that. And I get it, it's like more money involved, but like- like you said, it's like Canada's an immigrant country…"* - participant 11, male, Brazil
4) Championing family doctors to play a pivotal role in vaccine counseling and administration	Families thought that family doctors should take on a larger role in providing vaccine counseling, including seasonal vaccines, as well as providing reminders for when vaccines were due	*"There are two types of mandatory and non-mandatory [seasonal] so I never go for the non-mandatory [seasonal] ones because I don't know what they are putting, what- what, uh is for this vaccine because no one told me and if you will provide this information to the family doctor, a family doctor can guide all"* - participant 8, female, Hungary
	Having family doctors provide vaccines in Calgary was seen as a method to increase access to immunizations	*"Because I struggled … I don't have the car when I came [here] … so family doctor is just in front of my house but I cannot get the vaccine from him- doctor, I need to go some hospital in … three four kilometers. So if you would provide it to family doctor I think you don't have to worry about transportation because … always family doctors near you"* - participant 8, female, Hungary
5) Streamlining vaccine record tracking	Development of a vaccine equivalency table was seen as a method for families to keep track of vaccines received during their migration journey	*"We came from India, Australia, then Canada, then my daughter took the basic shot twice. She took in Australia and she took in Canada also, so I feel like the names should be same, so I can show my previous record and that should be carried here as well. So I've found there is no information on the names"* - participant 7, female, India
	An electronic vaccine passport or electronic certificates were identified as a method to facilitate vaccine record keeping	*"Back in Brazil… when the kid's born, you get this [vaccine record]. And you have everything, this is the schedule 'til like seven years old."* participant 11, male, Brazil
